# Effectiveness of structured, multidisciplinary long-term care for pediatric cancer survivors: protocol of the multicenter, randomized-controlled AELKI study

**DOI:** 10.1186/s13063-024-08377-2

**Published:** 2024-09-08

**Authors:** Hannah Schmidt, Katja Baust, Gabriele Calaminus, Lisa Hohls, Katharina Tetzner, Nicole Griech, Henrike Haugke, Hannah Baltus, Susanne Elsner, Alexander Katalinic, Hera Becker, Chirine Cytera, Judith Gebauer, Ann-Kristin Kock-Schoppenhauer, Anke Neumann, Christian Denzer, Michael M. Schündeln, Jörg Faber, Conny Sattler, Michael C. Frühwald, Anja Borgmann-Staudt, Anke Barnbrock, Markus Metzler, Gabriele Escherich, Inke R. König, Ingo Menrath, Thorsten Langer

**Affiliations:** 1 Clinic for Pediatric and Adolescent Medicine, University Hospital Schleswig-Holstein, Lübeck, Germany; 2 Center for Pediatrics, Bonn University Hospital, Bonn, Germany; 3 Institute for Social Medicine and Epidemiology, University of Lübeck, Lübeck, Germany; 4 Department of Internal Medicine 1, University Hospital Schleswig-Holstein, Lübeck, Germany; 5 IT Center for Clinical Research, University of Lübeck, Lübeck, Germany; 6 Department of Pediatrics and Adolescent Medicine, Section of Pediatric Endocrinology and Diabetology Hormone Center for Children and Adolescents, Ulm University Hospital, Ulm, Germany; 7 Division of Pediatric Hematology and Oncology, Department of Pediatrics III, University Hospital Essen, University of Duisburg-Essen, Duisburg, Germany; 8 Center for Pediatric and Adolescent Medicine, University Medical Center of Johannes Gutenberg University Mainz, Mainz, Germany; 9 Clinic for Pediatric and Adolescent Medicine, University Medical Center Schleswig-Holstein, Campus Kiel, Kiel, Germany; 10 Pediatrics and Adolescent Medicine, University Medical Center Augsburg, Swabian Children’s Cancer Center, Augsburg, Germany; 11 Department of Pediatrics With Focus On Oncology/Hematology, Charité Medical Center Berlin, Berlin, Germany; 12 Department of Pediatrics, Division of Hematology, Oncology and Hemostaseology, Goethe University, Frankfurt, Frankfurt/Main, Germany; 13 Clinic for Pediatrics and Adolescent Medicine, University Hospital Erlangen, Erlangen, Germany; 14 Department of Pediatric Hematology and Oncology, University Medical Center Hamburg-Eppendorf, Hamburg, Germany; 15 Institute of Medical Biometry and Statistics, University of Lübeck, Lübeck, Germany

**Keywords:** Cancer, Children and adolescent, Long-term care, Psychosocial, Multidisciplinary

## Abstract

**Background:**

In Germany, around 2.250 children and adolescents are diagnosed with cancer each year. Despite generally positive long-term survival rates, many patients must cope with late effects of the disease and its treatment. This highlights the need for a well-structured, long-term approach addressing both physical and mental health issues. Currently, the German healthcare system lacks such comprehensive structures. Our study aims to evaluate the effectiveness of a structured, multidisciplinary long-term approach compared to conventional “treatment as usual” (TAU).

**Methods:**

A prospective, multicenter study with ten pediatric university clinics in Germany will be conducted. The cluster-randomization takes place at the clinic level. Children and adolescents who completed their cancer treatment at least five years ago and their parents will be eligible to participate. While the control group (CG) receives TAU, the intervention group (IG) participates in a structured program. This program includes risk-based medical treatment and psychosocial interventions tailored to each patient’s individual needs within a two-month timeframe. The primary outcome is the improvement of self-efficacy. Secondary outcomes are satisfaction with health care, improvement of health-related quality of life (HRQoL), reduction of mental health problems, and improvement of transition readiness.

**Discussion:**

This approach has the potential to optimize the health care for individuals who survived cancer during childhood or adolescence. It addresses the challenges of overuse, underuse, and misuse of health care resources. By considering both medical and psychosocial factors and promoting increased self-efficacy, independent from parental involvement, it may facilitate a smoother transition to adult medicine and enhance adherence to lifelong aftercare. If proven successful, this approach will contribute to the integration of multidisciplinary strategies into standard healthcare practice.

**Trial registration:**

German Clinical Trials Register DRKS00029269. Registered on December 23, 2022.

## Administrative information

Note: the numbers in curly brackets in this protocol refer to SPIRIT checklist item numbers. The order of the items has been modified to group similar items (see http://www.equator-network.org/reporting-guidelines/spirit-2013-statement-defining-standard-protocol-items-for-clinical-trials/).

**Table:** 

Title {1}	Effectiveness of structured, multidisciplinary long-term care for pediatric cancer survivors: study protocol of the multicenter, randomized-controlled AELKI study
Trial registration {2a and 2b}.	German Clinical Trials Register, Registration number: DRKS00029269. Registered on December 23, 2022
Protocol version {3}	Protocol Version 1.0 on 1st June, 2023
Funding {4}	Funded by the German Innovation Fonds. Number: 01VSF21049
Author details {5a}	^1^ Clinic for Pediatric and Adolescent Medicine, University Hospital Schleswig–Holstein, Lübeck ^2^Center for Pediatrics, Bonn University Hospital, Bonn ^3^ Institute for Social Medicine and Epidemiology, University of Lübeck, Lübeck ^4^ Department of Internal Medicine 1, University Hospital Schleswig–Holstein, Lübeck ^5^ IT Center for Clinical Research, University of Lübeck, Lübeck ^6^ Ulm University Hospital, Department of Pediatrics and Adolescent Medicine, Section of Pediatric Endocrinology and Diabetology Hormone Center for Children and Adolescents, Ulm ^7^ Division of Pediatric Hematology and Oncology, Department of Pediatrics III, University Hospital Essen, University of Duisburg-Essen, Germany ^8^ University Medical Center of Johannes Gutenberg University Mainz, Center for Pediatric and Adolescent Medicine, Mainz ^9^ University Medical Center Schleswig–Holstein, Campus Kiel, Clinic for Pediatric and Adolescent Medicine, Kiel ^10^ University Medical Center Augsburg, Pediatrics and Adolescent Medicine, Swabian Children’s Cancer Center, Augsburg ^11^ Charité Medical Center Berlin, Department of Pediatrics with focus on Oncology/Hematology, Berlin ^12^ Department of Pediatrics, Division of Hematology, Oncology and Hemostaseology, Goethe University Frankfurt, Frankfurt/Main, Germany ^13^ University Hospital Erlangen, Clinic for Pediatrics and Adolescent Medicine, Erlangen ^14^ Department of Pediatric Hematology and Oncology, University Medical Center Hamburg-Eppendorf, Hamburg ^15^ Institute of Medical Biometry and Statistics, University of Lübeck, Lübeck
Name and contact information for the trial sponsor {5b}	German Aerospace Center Project Management Agency | Department of Health | Division Med. Genome Research, Systems Medicine Heinrich-Konen-Straße 1 | 53,227 Bonn | Germany
Role of sponsor {5c}	This study is funded by the German Innovationsfond. The study design, the data collection, the data analysis, the data interpretation, the manuscript writing, and publication decisions are conducted independently from the sponsor.

## Introduction

### Background and rationale {6a}

In Germany, around 2.250 children and adolescents (≤ 18 years) are diagnosed with cancer each year [1]. While long-term survival rates have improved over the last few years, many patients still experience long-term and/ or late effects resulting from the disease and its treatment [2–5]. These effects can affect various organ systems and range from mild and manageable limitations to potentially life-threatening complications, including subsequent neoplasms [2–5]. Additionally, cancer therapy can increase the risk for infertility, cardiac diseases, or metabolic syndrome [2–5]. The prevalence of long-term and/or late effects increases with the time passed since the primary cancer diagnosis and its treatment [6]. Consistently, patients may develop new, chronic diseases even decades after the completion of their cancer treatment. A study of American survivors of childhood and adolescent cancer demonstrated that by the age of 50 years, nearly all individuals (99.9%) experienced chronic health limitations, with 96% reporting severe health limitations [6]. In contrast, the age-matched control group exhibited an average cumulative incidence of 9.2% for all chronic health limitations and 2.3% for severe limitations [6]. Apart from physical health concerns, childhood and adolescent cancer survivors are subject to an elevated vulnerability to mental health issues [7] or reduced health-related quality of life (HRQoL) [8].

Recognizing and managing these long-term and/or late effects is essential for improving the overall health outcomes and HRQoL for cancer survivors across different age groups. To address these challenges, participation in a long-term, risk-adapted, structured healthcare is recommended [9]. Long-term follow-up in this cohort has resulted in benefits such as early detection of diseases and reduced hospitalizations [9]. Additionally, these patients exhibit improved disease awareness, enhanced health-related self-efficacy [10], and a reduced risk of late complications [10, 11]. To address the diversity of potential long-term and/or late effects depending on the cancer itself and the cancer treatment, a multidisciplinary approach involving general pediatricians, pediatric oncologists, and psychosocial professionals is necessary. This approach should be coordinated with general practitioners and pediatricians in outpatient care [12–14]. However, the German healthcare system often faces challenges in addressing long-term and late effects of pediatric cancer survivors [15]. Regional disparities and non-compliance with guidelines may impede the effectiveness of existing programs, alongside with a deficit in psychosocial care. These psychosocial concerns encompass aspects such as reintegration into the peer group, resuming school or training, and transitioning from child to adult health care [16]. Despite the importance of these topics, some institutions inadequately address them due to a lack of psychosocial personnel. Therefore, a nationwide, tailored long-term aftercare program is essential for pediatric and adolescent patients who are at least five years after the end of their acute oncological treatment.

### Objectives {7}

The present study is entitled “Effectiveness of structured, multidisciplinary long-term care for pediatric cancer survivors: study protocol of the multicenter, randomized-controlled AELKI study.” The aim of the AELKI study is to evaluate the impact of structured multidisciplinary long-term care for children and adolescents five years after the end of the cancer treatment compared to a conventional “treatment as usual” (TAU) condition.

### Trial design {8}

The study is designed as a prospective, multicenter, two-arm randomized-controlled trial (RCT) with two parallel groups. Randomization will be performed as cluster-randomization with a 1:1 allocation. The primary objective of this trial is to test the superiority of the intervention group compared to the control group receiving standard treatment. The primary endpoint is the improvement of patients’ and parents’ self-efficacy after two months (T1) and three months (T2) in the intervention group compared to the control group.

## Methods: participants, interventions, and outcomes

### Study setting {9}

This study provides long-term follow-up care to a cohort of patients who have completed their acute oncology treatment at least five years ago. The study will be conducted across ten pediatric university clinics in Germany, including those located in Augsburg, Berlin, Bonn, Erlangen, Essen, Frankfurt, Hamburg, Kiel, Mainz, and Ulm.

### Eligibility criteria {10}

The inclusion and exclusion criteria for participants in this study are as follows:

Inclusion criteria:

1. Children and adolescents ≤ 18 years and at least one parent/caregiver
2. Acute cancer treatment was completed at least five years ago
3. Digital informed consent

Exclusion criteria:

1. Young adults > 18 years
2. Acute cancer treatment
3. Insufficient understanding of the German language
4. No digital informed consent

### Who will take informed consent? {26a}

Study nurses and physicians at the participating pediatric university clinics will identify and approach potential participants. Ample time will be provided to individuals to contemplate their participation and voluntarily decide to join the study. Prior to participation, parental/caregiver consent will be obtained through a digital informed consent (“opt-in”). Moreover, all patients aged 16 years and older will also undergo a digital informed consent process.

### Additional consent provisions for collection and use of participant data and biological specimens {26b}

This study will not involve the collection of biological specimens.

## Interventions

### Explanation for the choice of comparators {6b}

The control condition selected for this study is conventional TAU, which represents the standard medical care received by patients in real-world settings. By comparing risk-adapted guideline-based medical care along with psychosocial support to TAU alone, the study aims to evaluate the potential additional benefit of the new treatment in comparison to the existing standard care.

## Intervention description {11a}

### Screening

A local study nurse will screen potential participants in the intervention and control groups based on predefined criteria (see the “Eligibility criteria {10}” section). Eligible individuals will be contacted by mail or phone, providing detailed study information. Prior to participating, all individuals will give informed consent during the clinic’s waiting time before their long-term follow-up appointment.

### Description of the intervention

After providing informed consent, participants in both the intervention group and control group will engage in a tablet-based survey to evaluate primary and secondary outcomes during the clinic’s waiting time.

Participants in the control group receive conventional TAU, while participants in the intervention group receive a structured multidisciplinary intervention. This includes risk-based medical care and additional psychosocial support within the clinic.

#### Risk-based medical care

In the intervention group, physicianss conduct a structured risk stratification in advance. Since there was no specific risk assessment tailored to childhood and adolescence at the study’s initiation, we utilized the existing risk stratification developed for adults [17] with minor adaptations for suitability in this context. Childhood cancer survivors (CCS) are classified into three risk groups (RG) based on their individual risk of developing late effects due to cancer diagnoses and treatments. The low-risk group includes patients with surgeries only (excluding brain tumor patients), patients with acute lymphoblastic leukemia (ALL) and chemotherapy alone (without radiotherapy), and patients with retinoblastoma. The moderate-risk group comprises patients with chemotherapy (excluding ALL with chemotherapy and retinoblastoma) and patients with both chemotherapy and surgery. The high-risk group includes patients who underwent radiotherapy or allogenic stem cell transplantation. Regardless of their risk group, it is recommended that all patients in the study have at least annual, long-term follow-up appointments.

#### Psychosocial support

Before the initiation of the study, discussions with experts in this research field were conducted to identify typical psychosocial topics that families often raise in the oncological aftercare, such as questions about the transition process, health-promoting behaviors (nutrition, physical activity), the prevention of potential morbidities, and the management of the siblings’ needs. In response, materials and flyers addressing these topics were developed in accordance with recently published PanCareSurFup recommendations [18]. The psychosocial intervention is not standardized but tailored to each patient’s needs. Typically, up to four contacts with the psychosocial team over the next two months will be offered, with the frequency determined by individual needs. These sessions can take place in the clinic or via videoconference to ensure the accessibility and a low-threshold approach.

### Criteria for discontinuing or modifying allocated interventions {11b}

There are no predefined criteria for discontinuing or modifying the allocated interventions. The participation in this study is voluntary and can be terminated at any time.

### Strategies to improve adherence to interventions {11c}

The interdisciplinary project teams in Lübeck, Bonn, and Hamburg meticulously developed both medical and psychosocial interventions before the study commenced. For both intervention and control groups, study nurses received comprehensive online training, along with standard operating procedures (SOP´s) for patient documentation using the CentraXX database [19].

#### Only intervention group

Physicians in the intervention group engaged in workshops focused on the iterative development and guideline-compliant risk stratification in long-term follow-up care. Currently, bi-weekly online meetings provide a platform for the physicians to address practical challenges and ensure quality assurance. The psychosocial team in the intervention group underwent a one-day training workshop. Currently, the psychosocial team meets twice a month in intervention groups for ongoing training and discussions on challenges in practice.

#### Only control group

Currently, physicians and study nurses in the control group participate in bi-weekly online meetings to discuss potential challenges during the study and enhance adherence to study participation.

### Relevant concomitant care permitted or prohibited during the trial {11d}

Implementing structured, multidisciplinary long-term care or usual care will not permit additional care other than the usual care or the structured multidisciplinary intervention which includes risk-based medical care and additional psychosocial support within the clinic for pediatric cancer survivors. Medical follow-ups, for example, due to diagnostic results, will be performed as needed both in the structured multidisciplinary intervention and in the usual care.

### Provisions for post-trial care {30}

All participants who take part in both follow-up surveys will receive a voucher of EUR 25 as an incentive. The follow-up surveys take place after two months (T1) and after three months (T2).

## Outcomes {12}

### Primary outcome

#### Self-efficacy 

To measure the self-efficacy of children and adolescents aged 12 years and older and the self-efficacy of the parents, an adoption of the General Self-Efficacy Scale, German Version [20] will be used. The General Self-Efficacy Scale measures the extent to which individuals believe they can influence events in their lives and overcome obstacles. It consists of ten items rated on a four-point Likert scale, with higher scores indicating a stronger belief in one’s own ability to handle challenges and achieve success. To assess self-efficacy in the context of long-term follow-up care, we have added the following instruction for patients, which is also adapted for use as an instruction in the proxy assessment by parents: “You have overcome a cancer diagnosis and have been in follow-up care for some time. Today, we are asking you questions because we are interested in how you handle new situations related to your health. As you answer the following questions, please think about the topics of ‘health’ and ‘health care’ and how well you manage situations associated with these topics.”

### Secondary outcomes

#### Satisfaction with health care

The perceived satisfaction with health care will be assessed with the Health Care Satisfaction module, a proxy report by parents up to 18 years old [21]. This module is part of the broader Pediatric Quality of Life Inventory (PedsQL) measurement system, which evaluates health-related quality of life in children and adolescents. It comprises 24 items with a 4-Point Likert scale organized into 6 subscales, each focusing on a different aspect of health care satisfaction, such as “information,” “inclusion of the family,” “communication,” “professional competence,” and “emotional needs.” Additionally, patients aged 12 years and older will complete an adaptation of the German short form of the Youth Health Care measure—Satisfaction, Utilization, and Needs (YHC-SUN) [22]. This version consists of 7 items organized into three subscales: satisfaction, utilization, and needs.

#### HRQoL

To assess the HRQoL, the core module from the PedsQL will be used. Parents will provide proxy reports for children up to 18 years old, while individuals aged 12 and older will complete self-reports [23]. The module consists of 23 items, which evaluate HRQoL across four domains: physical, emotional, social, and school functioning. Responses are rated on a five-point Likert scale.

#### Mental health problems

Mental health issues will be assessed using the German version of the Strengths and Difficulties Questionnaire (SDQ) [24]. Parents will provide proxy reports for children up to 18 years old, while individuals aged 12 and older will complete self-reports. The questionnaire is a widely used tool for assessing mental health and behavioral problems in children and adolescents. It includes 25 items with a 3-point Likert scale divided into 5 subscales (emotional symptoms, conduct problems, hyperactivity-inattention, peer relationship problems, and prosocial behavior), each designed to measure different aspects of a child’s emotional and behavioral functioning.

#### Transition readiness

An adaption of the Transition Readiness Assessment Questionnaire (TRAQ) GV 15 will be used to assess a young person’s readiness to transition from pediatric to adult health care [25] as a self-report from 14 years onwards. With its 15 items on a five-point Likert scale, it evaluates various aspects of transition readiness, including the ability to manage health care needs independently, and the ability to effectively communicate health needs and concerns with health care providers.

All questionnaires to assess the primary and the secondary endpoints have been validated for reliability and validity and are widely used in both research and clinical settings. Besides, we recorded sociodemographic data in line with the population-based German KIGGS study [26].

### Participant timeline {13}

**Fig. 1:**
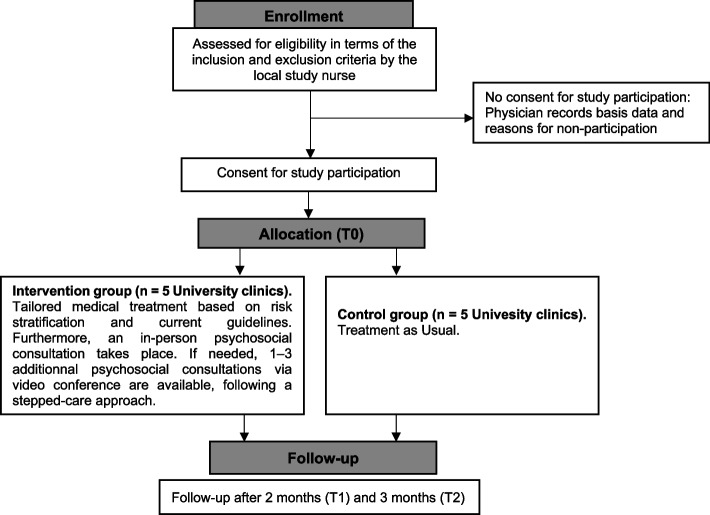
Participant timeline

The participant timeline is depicted in Fig. 1

### Sample size {14}

In the sample size calculation, we use the following assumptions: we expect the standardized effect of intervention versus control on self-efficacy in parents to be medium with a delta of 0.3 based on effect sizes obtained in comparable cohorts assessing similar outcomes. For example, a meta-analysis by Lundahl et al. [27] showed modest advantage of brief motivational interventions in enhancing health behavior and self-efficacy among various medical care settings. We aim for a power of 80% and account for a loss to follow-up of 10%. We assume an intra-cluster correlation (ICC) of 0.022 [28]. Taking varying cluster sizes into account, patients from at least *N* = 10 pediatric university clinics with a total of at least 160 participants have to be included in the study.

### Recruitment {15}

Each of the ten participating pediatric university clinics is responsible for recruiting its own participants. The recruitment period is 24 months. Prior to the recruitment, each clinic provided the project management team their average number of patients per month. On average, we expect 160 patients to be recruited in 24 months in each clinic. If recruitment difficulties arise, the recruitment period might be extended.

## Assignment of interventions: allocation

### Sequence generation {16a}

The cluster-randomized controlled, multicenter study encompasses ten participating pediatric university clinics, equally divided into five intervention and five control centers. The cluster-randomization was conducted centrally by the Institute of Medical Biometry and Statistics, University of Lübeck. We used a covariate-constrained cluster-randomization as implemented in R (package cvcrand, version 0.1). The variables experience of the center and number of expected families recruited per month were used as covariates.

### Concealment mechanism {16b}

The study site allocation was delayed as long as possible to ensure that sites had signed agreements and contracts to participate: final study sites had been identified, ethics approval obtained, and the study launch confirmed.

### Implementation {16c}

The result of the cluster-randomization was communicated to all participating pediatric university clinics in March 2023. All families within one center will receive the same intervention. To ensure commitment to the study, the cluster-randomization result was disclosed only after obtaining either a bilaterally signed collaboration agreement or a letter of intent from the participating clinics, regardless of the randomization outcome.

## Assignment of interventions: blinding

### Who will be blinded {17a}

Given the nature of the intervention, blinding of the patients and their parents is not possible. The data analysts will be blinded during the analyses.

### Procedure for unblinding if needed {17b}

A procedure for unblinding is not needed.

## Data collection and management

### Plans for assessment and collection of outcomes {18a}

At the baseline assessment (T0), the primary and the secondary outcome parameters will be assessed in the waiting time before the appointment at the clinic. Additionally, in the baseline assessment (T0), sociodemographic parameters are assessed with standard items based on the German Health Interview and Examination Survey for Children and Adolescents (KiGGS) survey, which is a representative long-term study on the health of children, adolescents, and young adults in Germany, conducted by the Robert Koch Institute [26]. The data assessment takes place via a tablet.

After two months (T1) and after three months (T2), all families receive a mail with links to the follow-up assessment. To increase the follow-up compliance, all families who do not take part in the follow-up assessment within one week receive a reminder by telephone and email.

### Plans to promote participant retention and complete follow-up {18b}

Participation in the intervention requires attending a counseling session with a member of the psychosocial team. Subsequent counseling appointments are optional and can be conducted either in person at the clinic or through online meetings, providing flexibility and increasing the likelihood of continued participation. To ensure the likelihood of complete follow-ups, participants receive a voucher of EUR 25 when they complete all follow-ups. Additionally, they receive up to two reminders via telephone and email if they do not respond within two weeks.

### Data management {19}

The primary outcome parameter, secondary outcome parameters, and self-reported sociodemographic data of patients and parents will be collected within the clinic setting using a tablet-based survey conducted through LimeSurvey (https://www.limesurvey.org). Once the survey via LimeSurvey is completed, an automatic routing mechanism leads the family to the SoSci Survey (https://www.soscisurvey.de), where their contact information is recorded. Reminders for follow-up surveys are sent to parents via email. Furthermore, physicians and study nurses collect patient medical data using CentraXX. The data are regularly checked for plausibility and completeness by the project management team in Lübeck. The data are protected, and access to the data is only possible with personal access. This procedure was devised in collaboration with the data protection officer in Lübeck and synchronized with all 10 clinics.

### Confidentiality {27}

For the cluster-randomized part of the study, participants will be assigned a unique ID upon giving informed consent, and all collected data will be stored under this ID. The central database in Lübeck will house these data, accessible externally by all study partners through CentraXX. Paper-based documentation forms will be archived by the study clinic for each participant for 10 years, adhering to the medical records retention period. Electronically stored medical data will be archived following the epidemiological practices outlined by the German Working for Epidemiology (German Acronym DAE) in 2004 for a minimum of 10 years. Participants can withdraw from the study at any time without any disadvantages, and in such cases, no further data will be collected. However, data processing that occurred before withdrawal will remain, unless the participant specifically requests data deletion along with withdrawal. Data deletion is only possible for non-anonymized data.

### Plans for collection, laboratory evaluation, and storage of biological specimens for genetic or molecular analysis in this trial/future use {33}

Not applicable. No biological specimens will be collected.

## Statistical methods

### Statistical methods for primary and secondary outcomes {20a}

For the primary objective regarding the primary efficacy endpoint, the primary hypothesis will be tested using a permutation test adjusted for cluster-level and subject-level covariates in the full analysis set, comprised of all participants who started the intervention. Details will be specified in the statistical analysis plan. The results will be complemented by estimates of the treatment effect using an adequate model that takes intra-cluster correlation into account (e.g., a linear mixed model or generalized estimating equations). As secondary objectives, we use the same model approach in the full analysis set to analyze the differences in the secondary efficacy endpoints.

A sequential testing procedure will be used to maintain a family-wise type I error of 0.05 for primary and secondary endpoints. If the primary endpoint is statistically significant at alpha = 0.05, the secondary efficacy endpoints will be tested. Hence, positive results on secondary endpoints can be interpreted inferentially only if a treatment effect is shown on the primary endpoint (gate-keeping). For all tested secondary endpoints, the significance level will be adjusted for multiple testing. The preliminary analysis plan is available upon request. The comprehensive analysis plan is currently being written and will be accessible in the German Clinical Trials Register once completed (https://drks.de/search/en/trial/DRKS00029269).

### Interim analyses {21b}

Not applicable, no interim analyses are planned.

### Methods for additional analyses (e.g., subgroup analyses) {20b}

Subgroup analyses will be specified in the statistical analysis plan and will include subgroups stratified by tumor recurrence and experience of other major diseases or life events during the course of the intervention. Sensitivity analyses of the primary endpoint will include variations in the handling of missing data. Furthermore, the primary endpoint will be evaluated in the intention-to-treat (ITT) and per-protocol (PP) populations. Further sensitivity analyses include the analysis of the secondary endpoints in the ITT and PP populations.

### Methods in analysis to handle protocol non-adherence and any statistical methods to handle missing data {20c}

If a participant declines study participation, the attending physician notes baseline data (age, gender, cancer diagnosis, and reason for declining participation) in a list and sends the consented baseline data to Lübeck every month. For missing values in the follow-up assessments, we will conduct multiple imputations.

### Plans to give access to the full protocol, participant-level data, and statistical code {31c}

The statistical analysis plan is available on request. Due to ethical and legal considerations and in line with the informed consent form, we cannot provide access to data at the participant level.

## Oversight and monitoring

### Composition of the coordinating center and trial steering committee {5d}

The project management of the study, headquartered in Lübeck, is responsible for supervising and tracking the study’s advancement. The team consists of physicians and psychologists and receives assistance from the Institute of Social Medicine and Epidemiology as well as the Institute of Medical Biometry and Statistics. Furthermore, the study involves active participation from multiple study centers, including Augsburg, Berlin, Bonn, Erlangen, Essen, Frankfurt, Hamburg, Kiel, Mainz, and Ulm.

### Composition of the data monitoring committee, its role and reporting structure {21a}

The intervention development and statistical analyses will be carried out by separate institutes, ensuring independent processes. The data monitoring protocol and planned statistical analyses were established prior to enrolling the first patient in the study. Additionally, the trial was registered in the German Clinical Trials Register.

### Adverse event reporting and harms {22}

While the overall risk of the intervention is considered low, it is important to acknowledge that (re-) exposure to the cancer experience during psychological counseling may cause short-term psychological stress. If this occurs, the patient and/or parent will be referred to local support services, such as outpatient psychiatric or psychotherapeutic care. Any questions, events, challenges, or similar issues that arise can be discussed with the physicians in the biweekly online meeting or at any time in between by email or telephone call with the project team. Unplanned deviations and possible solutions are documented in detail by the project team. In case of unexpected severe harm that is attributable to the intervention, the Lübeck Ethics Committee and the funding organization will be informed.

### Frequency and plans for auditing trial conduct {23}

Each participating site will implement internal quality management measures to ensure the proper conduct of the study, accurate data collection, thorough documentation, and completion of case report forms. A tailored quality management plan and report will be created to evaluate each site’s performance. Prior to enrolling participants, site research staff will undergo comprehensive training on the study protocol, following standard practices. The project team in Lübeck and Bonn will monitor the quality control and reliability of screening, baseline data collection, and follow-up. Monthly study videoconferences will be held to share the results with site research coordinators and partners. If any quality assurance issues are identified, the site principal investigators and project managers will collaborate to address them.

### Plans for communicating important protocol amendments to relevant parties (e.g., trial participants, ethical committees) {25}

Any relevant changes of the intervention and the evaluation will be submitted to the ethics committees for approval. If requested, participants will also be informed.

### Dissemination plans {31a}

We will publish the results of the AELKI study in peer-reviewed journals and present them to the scientific community. We will also inform patients via relevant journals and our own newsletter, as well as via patient representative groups. Besides, we will update the trial register. Since we did not inform the patients at the beginning of the study, we do not intend to publish the datasets analyzed during the current study because of our local data protection rules.

## Discussion

The study aims to establish and evaluate a structured multidisciplinary long-term care approach for children and adolescents, at least five years after the end of the regular cancer treatment. This approach focuses on the early detection and treatment of potential therapy-associated long-term and/ or late effects, considering both somatic and psychosocial factors. By employing evidence-based guideline recommendations [19] and utilizing risk stratification and informative materials for a successful transition, the study aims to provide high-quality healthcare to affected individuals, addressing the gap in their care and avoiding unnecessary treatment. Regular check-ups are expected to reduce long-term morbidity by enabling early treatment of potential late effects. Families who were previously unaware of their child’s risk for late effects will be informed and guided towards structured care. For adolescents’ transitioning from pediatric to adult medicine, this program offers the opportunity to be informed early about available healthcare options, facilitating a smoother transition into adult healthcare.

Beyond the potential benefits at the individual level, there are also potential implications for health economics. Currently, the positions of psychosocial workers within long-term oncological care at pediatric university hospitals in Germany are often not part of regular budget planning. Typically, they are funded exclusively through third party donations. If this trial proves that risk-based medical treatment and additional psychosocial support lead to enhancements in self-efficacy, satisfaction with the care, HRQoL, mental health problems, and transition readiness, it could significantly contribute to the funding of sufficient and multidisciplinary positions in the long-term care of pediatric cancer survivors.

## Trial status

The trial is registered on the German Clinical Trials Register (https://www.drks.de/DRKS00029269) with the registration number DRKS00029269. The current protocol is version 1.0 of 1 June 2023. The recruitment started on 1 July 2023. The approximate date of recruitment completion is 30 April 2025.

## Data Availability

Currently, the data are not accessible due to the ongoing recruitment process. The study findings will be disseminated through publication in a peer-reviewed journal and presentations at scientific conferences. There is an intention to provide access to the data for researchers upon request in justified cases. To facilitate this, a transparent protocol for granting anonymized access to the data is in the planning stages, ensuring compliance with data protection guidelines in justified instances.

## Abbreviations

RCT: Randomized-controlled trial
ITT: Intention-to-treat
PP: Per-protocol
HRQoL: Health-related quality of life
